# Recent Advances in Ex Situ Surface Treatments for Lithium Metal Negative Electrodes in Secondary Batteries

**DOI:** 10.3390/ijms26073446

**Published:** 2025-04-07

**Authors:** Paul Maldonado Nogales, Sangyup Lee, Seunga Yang, Soon-Ki Jeong

**Affiliations:** 1 Department of Future Convergence Technology, Soonchunhyang University, Soonchunhyang-ro 22-gil, Sinchang-myeon, Asan-si 31538, Chungcheongnam-do, Republic of Korea; maldonado@sch.ac.kr (P.M.N.); 20237450@sch.ac.kr (S.L.); tmddk1107@sch.ac.kr (S.Y.); 2 Department of Energy Engineering, Soonchunhyang University, Soonchunhyang-ro 22-gil, Sinchang-myeon, Asan-si 31538, Chungcheongnam-do, Republic of Korea

**Keywords:** lithium metal negative electrode, ex situ surface treatment, solid electrolyte interphase (SEI) stability, dendrite suppression, surface engineering, pretreatment techniques

## Abstract

Lithium metal negative electrodes are pivotal for next-generation batteries because of their exceptionally high theoretical capacity and low redox potential. However, their commercialization is constrained by critical challenges, including dendrite formation, volumetric instability, and the fragility of the solid electrolyte interphase (SEI). In this context, this review highlights the transformative potential of ex situ surface treatments, which stabilize lithium metal electrodes before cell assembly. Key advancements include inorganic and polymer-based coatings that enhance SEI stability and mitigate dendrite growth, three-dimensional host architectures that manage volumetric changes and improve lithium diffusion, and liquid-phase chemical modifications that enable uniform lithium deposition. These strategies are critically evaluated for their scalability, environmental sustainability, and long-term stability, paying particular attention to cost, complexity, and ecological considerations. In addition, their potential contributions to the development of advanced battery technologies are discussed, providing insights into pathways toward enhanced commercial viability. By synthesizing cutting-edge research and identifying unresolved challenges, this review provides a comprehensive roadmap for advancing safer, more efficient, and more durable lithium metal batteries, thereby bridging the gap between laboratory research and commercial adoption.

## 1. Introduction

Lithium exhibits unique properties that make it a promising material for use as negative electrodes in advanced battery systems. Its high theoretical specific capacity (3860 mAh g^−1^), low redox potential (−3.04 V vs. the standard hydrogen electrode), and lightweight gravimetric density (0.534 g cm^−3^) [1,2] make lithium a strong candidate for next-generation secondary batteries. These attributes hold substantial potential to improve the performance of next-generation systems such as lithium–sulfur (Li–S) and lithium–oxygen (Li-O_2_) batteries [3,4]. In addition, lithium metal has been explored as a potential negative electrode material for all-solid-state batteries, offering enhanced safety and energy density through the use of solid electrolytes. However, despite these advantages, lithium metal faces several critical challenges, particularly dendrite formation, volumetric instability, and the fragility of the solid electrolyte interphase (SEI), which directly impact battery safety, cycling stability, and overall efficiency.

Dendrites are tree-like structures resulting from uneven lithium deposition during charging that can penetrate the separator and cause internal short circuits, posing severe safety risks. In addition, the continuous deposition and stripping of lithium during cycling induces significant volumetric changes, leading to poor cycling stability, active lithium loss, and reduced overall battery efficiency and lifespan [5,6]. The SEI, which is typically fragile and prone to cracking during cycling, exposes fresh lithium to the electrolyte, thereby intensifying side reactions and further destabilizing the electrode. Thus, researchers have concentrated on developing strategies to stabilize lithium metal electrodes and mitigate these risks. For this purpose, an ideal SEI should exhibit high ionic conductivity, electronic insulation, chemical and mechanical stability, and a uniform nanometer-scale thickness [2,7,8], thereby forming the foundation for a wide range of stabilization approaches.

To address these issues, a range of strategies have been proposed to stabilize lithium metal negative electrodes in secondary batteries, including fluorinated electrolyte additives [5,6], solid-state electrolytes [9,10], super-concentrated electrolytes [11], engineered SEIs [12,13], structured negative electrodes [14,15], and separator reinforcement [16,17]. Although in situ methods rely on dynamic SEI formation during cycling, ex situ methods enable the controlled preformation of stable protective layers under optimized conditions. For example, ex situ surface pretreatments, such as vapor deposition or liquid-phase chemical treatments, provide mechanistic insights into SEI formation and lithium diffusion processes while aligning with scalable approaches for practical applications [18].

Building on these advancements, this review systematically examines ex situ surface treatments for lithium metal negative electrodes and emphasizes their contributions to SEI stability and lithium diffusion. In addition, these strategies are critically evaluated based on their environmental sustainability and long-term stability while addressing key challenges such as cost and manufacturing complexity. By narrowing the focus to ex situ approaches, this review highlights recent innovations while grounding future advancements in robust scientific principles and sustainability considerations. The remainder of this review is structured as follows: Section 2 categorizes recent advances in ex situ surface engineering strategies, including SEI formation techniques and protective layer modification. Section 3 synthesizes these findings, offers conclusions and future perspectives that highlight unresolved challenges, and proposes pathways to develop safer, more efficient, and more durable lithium metal batteries.

## 2. Ex Situ Surface Engineering Strategies for Lithium Metal Negative Electrodes

Ex situ surface treatments for lithium metal negative electrodes are applied before cell assembly, distinguishing them from in situ modification strategies that operate dynamically during battery cycling. These approaches offer a promising pathway to stabilizing the SEI layer and enhancing lithium-ion diffusion, thereby effectively addressing persistent challenges such as dendrite formation and volumetric changes during cycling. Unlike in situ strategies, which rely on spontaneous SEI formation via electrolyte decomposition, ex situ methods enable pre-engineered, uniform protective layers under controlled conditions—offering improved reproducibility and design flexibility. Recent advancements have emphasized the precise engineering of structured SEI layers under controlled environments, specifically for pretreating lithium metal surfaces. For example, vapor deposition and liquid-phase treatments have been employed to create uniform, durable SEI layers that allow greater control over the electrochemical properties of the lithium surface. These techniques have significantly contributed to the development of safer and longer-lasting battery systems, thereby advancing both performance and reliability.

### 2.1. SEI Formation and Pretreatment Techniques

Recent advancements have demonstrated that ex situ pretreatment methods significantly improve the performance of lithium metal negative electrodes by forming stable and robust SEI layers before battery assembly. These treatments effectively create a durable protective barrier on the lithium metal surface, minimize undesirable side reactions with the electrolyte, enable uniform lithium-ion deposition, and significantly mitigate degradation during electrochemical cycling. Key approaches include inorganic coatings, which offer robust chemical stability, and polymer-based protective layers, which provide mechanical flexibility and resilience against volumetric changes. While inorganic coatings excel at maintaining chemical inertness under harsh electrochemical conditions, polymer-based coatings complement these properties by accommodating volume fluctuations, thereby improving cycling performance and durability.

#### 2.1.1. Metallic and Inorganic Coatings for SEI Stabilization

**Figure 1:**
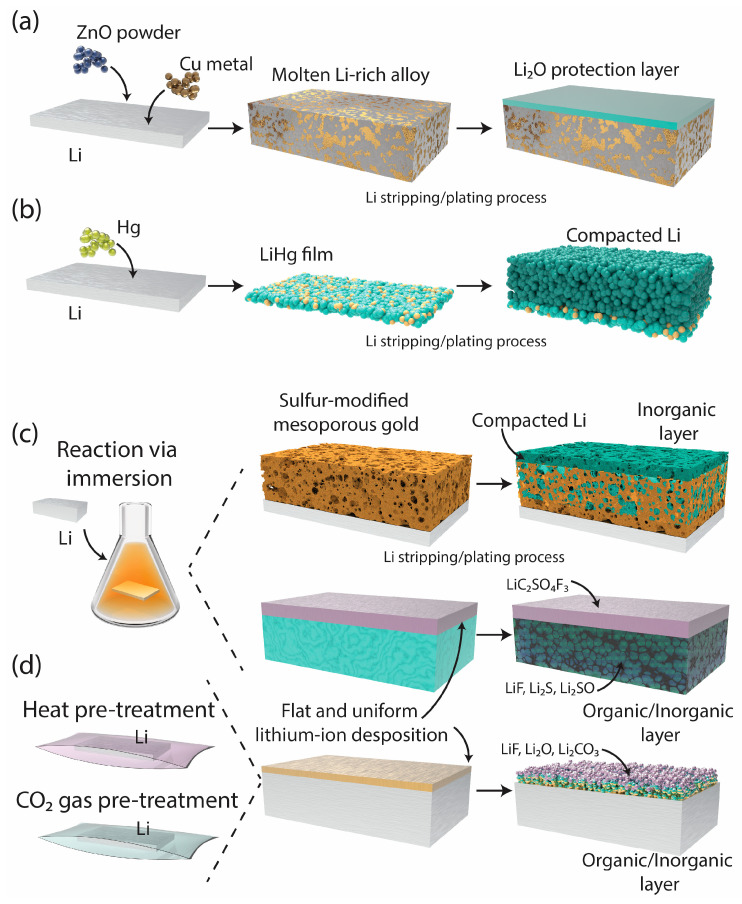
A schematic of metallic and inorganic coating pretreatments applied to lithium surfaces, redrawn based on data from previous studies: (**a**) a copper–zinc alloy forming a molten Li-rich protective layer for enhanced structural rigidity and lithium-ion transport [19]; (**b**) a mercury-based alloy creating a Li-rich layer with high atomic mobility to promote uniform lithium deposition [20]; (**c**) a sulfur-modified mesoporous gold layer formed via immersion to improve lithium-ion affinity and deposition uniformity [21,22]; and (**d**) multicomponent organic and inorganic layers produced using heat and CO_2_ gas reactions to strengthen SEI and reduce interfacial resistance [24,25].

Inorganic and metallic coatings have emerged as promising strategies to improve the performance of lithium metal electrodes by improving surface chemistry and mechanical strength. These coatings promote high thiophilicity, forming robust interfacial layers that facilitate uniform lithium-ion transport and deposition. Figure 1 summarizes various metallic and inorganic coating approaches for SEI stabilization, highlighting strategies such as alloy-based protective layers (a, b), sulfur-modified mesoporous films (c), and multicomponent organic/inorganic coatings (d). Early SEI engineering efforts involved the ex situ incorporation of transition metals to improve the chemical stability of electrode surfaces. Subsequent advancements have targeted mixed-component SEIs and carbonate-rich films, thereby overcoming the inherent challenges of lithium metal batteries through innovations in material design and chemical processing.

Building on these foundational insights into SEI stability, recent studies have focused on alloy systems such as Cu–Zn and Li–Hg, which significantly improve lithium nucleation and deposition uniformity. Researchers have investigated various metals, including copper (Cu), zinc (Zn), sulfur (S), gold (Au), and mercury (Hg). For example, Yi et al. demonstrated that a ternary alloy framework composed of these metals enhances thiophilicity by providing favorable nucleation sites for lithium ions (Figure 1a) [19].

This improved nucleation behavior is strongly influenced by the intrinsic nucleation energy of the substrate. For instance, density functional theory studies have reported that lithium exhibits a lower nucleation barrier on gold (∼0.06 eV) and zinc (∼0.09 eV) compared to copper (∼0.17 eV), favoring more uniform lithium plating. Mercury, in the form of Li–Hg amalgams, presents an even lower nucleation energy due to its liquid nature and high surface mobility, enabling dendrite-free deposition under high areal capacities. These differences highlight the importance of substrate selection in engineering efficient lithium metal interfaces. However, their findings revealed a trade-off—although increasing the ZnO content within the alloy enhances structural rigidity, it also increases the matrix complexity, thereby limiting lithium-ion mobility and increasing internal resistance. This trade-off highlights a persistent challenge in SEI engineering—balancing mechanical strength and ion transport for optimal battery performance. To address this issue, researchers have explored alloy systems with high atomic mobility, such as the Li–Hg amalgam developed by Li et al. The high diffusion coefficient of mercury facilitates rapid atomic migration, enabling compact lithium deposition and overpotential reduction (Figure 1b) [20]. This dynamic interface is effective under high-capacity conditions, maintaining dendrite-free growth at 55 mAh cm^−2^. However, the toxicity and environmental risks associated with mercury highlight the need to identify safer and more sustainable alternatives with similar diffusion properties.

In response to the challenges posed by single-metal systems, researchers have explored chemically modified substrates as an alternative. Na et al. investigated sulfur-modified mesoporous gold films to improve the consistency of lithium removal and plating (Figure 1c). Sulfur enhances lithium-ion affinity, promoting uniform lithium deposition and stabilizing the SEI during cycling [21]. Nevertheless, reliance on a single compound, such as sulfur, poses challenges in achieving a balance between chemical stability and ionic conductivity. Combining materials can effectively address these limitations, as demonstrated in subsequent studies. Building on these findings, Pang et al. introduced multicomponent SEIs composed of LiF, Li_2_S, and Li_2_SO_3_. Their study demonstrated that LiF ensures chemical stability, whereas Li_2_S and Li_2_SO_3_ enhance ionic conductivity, collectively improving lithium-ion transport. The synergistic interaction between these components creates a uniform ionic flux at the electrode interface, mitigates dendrite formation, and enhances cycling stability [22]. The shift from single-component to multicomponent SEIs addresses the trade-offs between stability and conductivity, contributing to the development of more efficient and durable lithium metal batteries. However, precise parameter control during carbonate integration remains a critical challenge, highlighting the need for further optimization.

In addition to synthetic and biomimetic strategies, protein-based biopolymers have emerged as sustainable ex situ treatments to stabilize lithium metal electrodes. For example, Wu et al. used sericin, a silk-derived protein, to form an ion-conductive SEI that suppressed dendrites and enhanced cycling at high current densities [3]. Similarly, Wang et al. applied heat-treated zein (from corn), which exposed polar groups and improved interface wettability, enabling compact SEI formation and uniform Li plating [23]. These bio-derived polymers not only align with environmentally sustainable goals but also demonstrate compelling electrochemical benefits, supporting their potential as scalable ex situ SEI precursors for lithium metal protection.

Although multicomponent SEIs address the trade-offs between stability and conductivity, carbonate-based coatings offer complementary benefits by enhancing SEI robustness. Building on the benefits of carbonate compounds (Figure 1d), Nogales et al. employed heat treatment processes to uniformly integrate Li_2_CO_3_ within the SEI layer [24]. Their findings revealed that heat treatment reduces interfacial resistance and enhances mechanical integrity, significantly improving battery performance over extended cycles. However, achieving consistent SEI compositions across various battery systems requires precise control of the heat treatment parameters, such as temperature and duration. Complementing these thermal approaches, Kim et al. introduced a pretreatment method using CO_2_ gas to form a carbonate-rich native film on the lithium surface. This alternative method simplifies the process by using naturally abundant CO_2_ gas, thereby producing an SEI with enhanced electrochemical properties. The resulting SEI provides a stable ion-conductive interface, thereby mitigating side reactions and suppressing dendrite growth [25]. These environmentally friendly treatments align with the principles of sustainable battery production and demonstrate the potential of naturally abundant compounds to improve the performance of lithium metal negative electrodes. Emerging evidence indicates that controlling the lithium metal surface prior to cycling significantly mitigates the formation of electrically isolated ‘dead’ lithium, enhancing coulombic efficiency and interfacial stability through more uniform SEI evolution and lithium deposition dynamics [26,27,28].

As illustrated in Figure 1, these strategies range from alloy frameworks (a, b) to chemically modified substrates and carbonate-based layers (c, d), highlighting the diverse approaches employed in SEI engineering. Collectively, these studies demonstrate how inorganic and metallic coatings have progressed from simple single-component layers to complex multimaterial systems, effectively addressing challenges related to stability, conductivity, and scalability. However, their inherent rigidity limits their ability to accommodate the volume changes that occur in lithium during cycling. To overcome these mechanical limitations, researchers have shifted their focus to polymer-based coatings. Unlike inorganic and metallic coatings, polymer-based coatings offer unique advantages such as elasticity, flexibility, and self-healing properties. These characteristics are crucial for maintaining SEI integrity under mechanical stresses, ensuring long-term cycling stability, and improving overall battery performance.

#### 2.1.2. Polymer-Based Protective Layers

Polymer coatings provide versatile and innovative solutions for protecting lithium metal electrodes through ex situ pretreatments. By combining mechanical flexibility with electrochemical stability, these coatings can adapt to volume fluctuations, suppress dendrite growth, and preserve SEI integrity. Researchers have explored various polymer-based strategies to enhance the durability of lithium negative electrodes. These investigations reveal how tailored polymer compositions can improve electrolyte compatibility, interfacial resistance, and overall battery performance.

**Figure 2:**
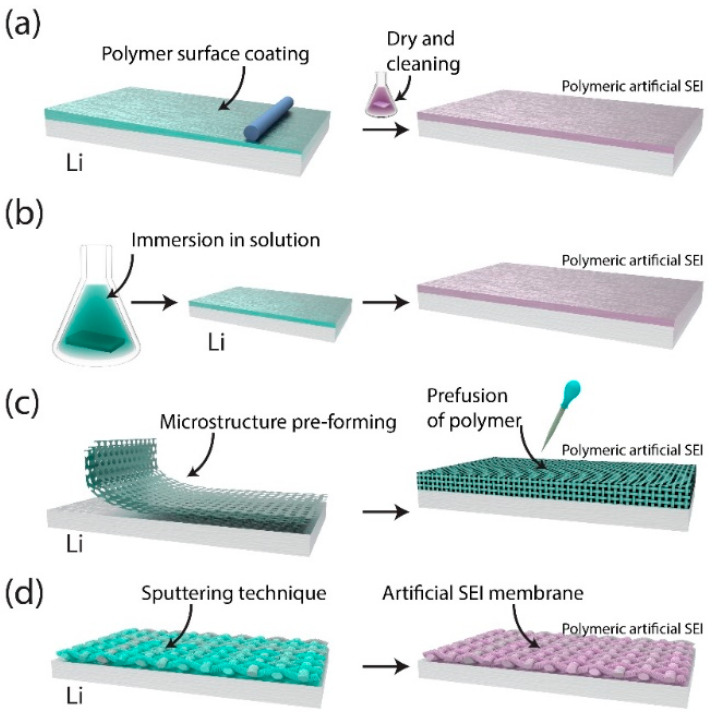
A schematic of surface processing strategies for polymer coatings applied to lithium metal negative electrodes, redrawn based on data from previous studies: (**a**) polymer surface coating, followed by drying and cleaning, forming a polymeric artificial SEI designed to improve chemical stability [29]; (**b**) immersion in a polymer-containing solution, creating a polymeric artificial SEI aimed at suppressing dendrite growth [30,31,32,33]; (**c**) microstructure preforming to fabricate a polymeric artificial SEI with enhanced ionic conductivity and mechanical flexibility [34]; and (**d**) a sputtering technique to deposit a uniform polymer layer, forming an artificial SEI membrane for enhanced interfacial properties [35,36].

To further explore the adaptability of polymer coatings, researchers have developed composite polymer layers that combine mechanical strength with ionic conductivity (Figure 2). For example, Ahmed et al. developed a surface-treated composite polymer layer (STCPL) by blending polyacrylonitrile (PAN) with lithium bis(trifluoromethanesulfonyl)imide. Thus, the STCPL forms a stable, flexible SEI that accommodates mechanical stresses during cycling. By promoting uniform lithium-ion flux and reducing localized current densities, it effectively suppresses dendrite growth. However, Ahmed et al. noted that mechanical degradation over prolonged use remains a concern because the performance of the polymer layer diminishes after extended cycling [29]. Building on Ahmed et al.’s findings, Chen et al. and Liu et al. developed fluoropolymer-based SEI layers incorporating strong C–F bonds and inorganic additives (Figure 2a,b) [30,31]. Their copolymerization techniques produced robust polymer matrices, including poly (vinylidene fluoride) blended with LiF and LiNO_3_, resulting in dense, protective coatings. These fluoropolymers exhibit both high ionic conductivity and effective dendrite suppression. However, both research groups highlighted that achieving consistent copolymer sequences remains a challenge because polymerization process variations can significantly impact SEI performance. Therefore, ensuring reproducibility and scalability in manufacturing requires precise control of the synthesis parameters.

Expanding beyond composite polymer layers, further innovations have explored plasma polymerization techniques for creating chemically stable and flexible SEIs (Figure 2b). For example, Cao et al. performed the in situ plasma polymerization of polythiophene, thereby creating a flexible yet chemically stable layer on lithium [32]. This conductive interface promotes uniform Li^+^ deposition, significantly extending the life of the battery. Furthermore, plasma polymerization enables seamless integration with lithium surfaces, thereby reducing the interfacial resistance and ensuring longevity. Building on this approach, Naren et al. introduced a reactive polymer that chemically interacts with lithium, forming a stable SEI rich in Li–C and Li–N bonds [33]. These functional bonds improve both ionic conductivity and structural integrity, effectively balancing the mechanical and electrochemical properties. Together, these studies illustrate how tailored polymer coatings can serve as both physical barriers and ion-conductive interfaces, optimizing lithium-ion transport and improving overall battery performance.

Beyond advanced synthesis techniques such as plasma polymerization, biomimetic designs such as the triply periodic minimal surface (TPMS) architecture offer a unique perspective by incorporating structural inspiration from nature (Figure 2c). Ma and collaborators developed a poly (vinylidene fluoride-co-hexafluoropropylene) layer with a TPMS architecture inspired by sea urchins. This biomimetic design leverages the lightweight yet robust skeletal structures of sea urchins to achieve high mechanical strength while reducing the overall weight of the battery. By enhancing the energy density, the TPMS structure provides a notable advantage for high-performance applications [34]. In addition, the porous architecture facilitates continuous Li^+^ pathways, thereby improving ionic conductivity and minimizing capacity fade during extended cycles. Despite these promising attributes, Ma et al. emphasized the need for further investigation into the long-term performance and scalability of these designs. Key challenges include the high cost and complexity of manufacturing TPMS-based coatings, which require process optimization for commercial feasibility.

Complementing structural innovations such as TPMS, environmentally friendly designs emphasize sustainability alongside performance enhancements (Figure 2d). Song et al. developed a water-based SEI layer composed of biocompatible carboxymethyl guar gum and polyacrylamide. These biopolymers align performance improvements with sustainability goals by forming a flexible and adhesive layer that withstands mechanical stress and accommodates volume changes [35]. Song et al.’s work not only extends the battery cycle life but also demonstrates a reduction in the environmental footprint of lithium battery production by using renewable materials. This environmentally conscious approach provides a dual benefit by improving performance while reducing ecological impacts.

Although sustainability-focused designs are promising, challenges related to electrolyte compatibility remain critical for polymer coatings. For example, Kwon and Lee developed a lithium silicate–lithium phosphate composite layer that suppresses dendrite growth while enhancing ionic conductivity [36]. Although these results are promising, both groups identified the long-term stability of these modified interfaces as a critical area for further investigation, particularly under diverse operating conditions, including wide temperature ranges and varying cycling rates. Addressing these challenges is crucial for the practical application of polymer coatings in lithium metal batteries.

These studies have highlighted the potential of polymer coatings to address the key challenges associated with lithium metal negative electrodes, including dendrite growth, mechanical integrity, and electrolyte compatibility. By tailoring the chemical composition and structure of polymer layers, researchers can design SEIs that balance mechanical strength with ionic conductivity. The next steps involve refining these coatings to enhance lithium diffusion, better manage mechanical stresses caused by volume changes during cycling, and achieve scalable, reproducible production methods for commercial integration. Moreover, in situ techniques such as atomic force microscopy, X-ray photoelectron spectroscopy, scanning electron microscopy, Raman spectroscopy, and nuclear magnetic resonance have emerged as powerful tools to investigate the real-time evolution of SEI and lithium deposition behavior, helping to evaluate the effectiveness of protective polymer coatings [28,37,38,39].

Additionally, recent evidence suggests that carbon-rich polymer layers may contribute to the formation of hard LixCy phases during cycling [26,27], which have been linked to dendrite penetration through separators, an aspect that requires careful control in future coating designs. Accordingly, the selection of polymer coatings should carefully consider their thermal and electrochemical decomposition pathways to minimize unintended carbide formation.

### 2.2. Direct Material Manipulation Methods for Surface Modification

Structural modification is a key ex situ strategy for optimizing lithium-ion transport. These techniques include designing three-dimensional (3D) host architectures and altering the surface morphology of lithium. By promoting uniform lithium deposition, these modifications reduce localized current densities and alleviate mechanical stress caused by volume changes during cycling. These improvements enhance cycling stability and overall battery performance.

#### 2.2.1. Three-Dimensional Host Structures

The use of 3D host structures has emerged as an effective strategy for treating lithium metal negative electrodes. These structures provide a conductive and mechanically stable matrix that absorbs volumetric fluctuations without cracking, thereby facilitating uniform lithium-ion transport. By reducing localized stress and accommodating volumetric changes, key challenges such as dendrite formation and uneven lithium distribution can be mitigated. Thus, 3D host structures play a pivotal role in improving battery performance by minimizing capacity fade and extending the cycle life.

**Figure 3:**
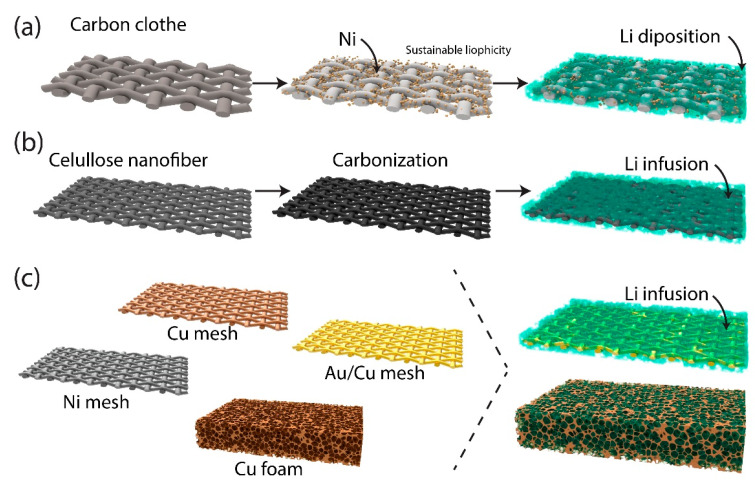
The fabrication of 3D host structures for lithium deposition using surface-pretreated materials, redrawn based on previous studies: (**a**) a carbon cloth coated with nickel to promote sustainable thiophilicity and uniform lithium deposition [40]; (**b**) cellulose nanofibers carbonized to form a highly conductive framework, aimed at enhancing lithium infusion and reducing dendrite growth [41]; and (**c**) various metal meshes, including copper, nickel, gold/copper, and copper foam, designed to provide structural support and promote uniform lithium infusion across the electrode surface [42,43,44,45].

To further enhance the capabilities of 3D host structures, researchers have explored diverse architectures and materials tailored for lithium deposition (Figure 3). Notable materials include anti-perovskite nitrides, which provide excellent ionic conductivity; copper phosphide nanowires, which are known for their mechanical stability; and thiophilic carbon hosts, which promote uniform lithium plating. For example, Huang et al. introduced NiCo nanocubes to create a robust 3D scaffold that combines high electrical conductivity with excellent mechanical strength (Figure 3a). This scaffold effectively manages volumetric expansion during cycling, thereby maintaining structural integrity and enabling consistent lithium-ion transport. By minimizing structural degradation over time, such durable architectures significantly improve battery performance and extend the operational life [40].

Expanding the scope of materials, alternative approaches, such as carbonized bacterial cellulose nanofibers, offer unique advantages for lithium metal negative electrodes (Figure 3b). The interconnected network of nanofibers provides abundant nucleation sites, thereby facilitating homogeneous lithium deposition and effectively suppressing dendrite growth [41]. This design creates synergy between the large surface area and strong lithium affinity, ensuring smooth ion pathways and reducing localized current densities. Thus, the risk of uneven lithium plating is significantly reduced, leading to an extended cycle life and enhanced battery stability.

A key strength of these 3D scaffolds is their ability to sustain performance over extended cycling (Figure 3c). Kim et al. and Shao et al. demonstrated significant improvements in cycle stability, achieving steady performance over hundreds to thousands of hours. Kim et al.’s 3D porous copper host, coated with thiophilic materials, enhances lithium-ion affinity and promotes uniform deposition [42]. Building on this foundation, Zheng et al. integrated a lithium–zinc alloy surface within a 3D host structure, further advancing the technology. The alloy forms a thiophilic interface that reduces the energy barrier for lithium nucleation, thereby facilitating uniform deposition at high current densities [43]. Similarly, Zeng et al. proposed a self-adapting electrochemical strategy using a 3D carbon host with a gradient of lithiophilic silver nanoparticles, pre-assembled onto ultrathin lithium foils. During cycling, lithium migrates into the host, mitigating volume changes and suppressing dendrite growth through bottom-up deposition. Although lithium redistribution occurs in situ, the scaffold is fabricated and integrated ex situ, qualifying it as a structural surface treatment [44]. Their system sustained battery stability for over 1900 h and exhibited superior performance in full-cell configurations. These findings demonstrate the potential of integrating 3D hosts and alloy interfaces to address dendrite growth challenges and meet scalability requirements under high energy demands.

Despite these advancements, scalability and manufacturing complexity continue to pose significant barriers to the widespread adoption of 3D host structures. Techniques such as electrodeposition, chemical vapor deposition, and templating methods excel in terms of precision but are challenging and costly to scale for mass production. In addition, the reliance on expensive raw materials, such as nickel and cobalt, further complicates commercialization efforts. Hui and Li emphasized the importance of evaluating the environmental impact of these processes and advocated for innovative strategies to reduce the ecological footprint associated with nanomaterial synthesis [45,46]. Addressing these concerns is critical for balancing precision with environmental responsibility and enabling scalable, eco-friendly solutions.

Although existing techniques face scalability challenges, alternative approaches such as porous copper meshes and additive manufacturing offer promising solutions by balancing cost and performance. For example, Kim et al. developed a porous copper mesh as a 3D host, offering a more cost-effective and scalable solution than nanomaterial-based hosts. Although this design lacks certain advanced features, further optimization can bridge the gap between high performance and manufacturability. In addition, recent advances in additive manufacturing have demonstrated the ability to fabricate complex, architected 3D scaffolds with precisely controlled geometry, porosity, and material composition. Techniques such as direct ink writing and stereolithography allow for the creation of mechanically robust, lithiophilic hosts that can accommodate volume changes and improve interfacial stability by promoting uniform lithium deposition [47,48]. These engineered structures can play a crucial role in stabilizing the SEI and enhancing lithium-ion transport. In addition to material choices, advancements in additive manufacturing present new opportunities for the production of complex 3D structures at scale. These techniques combine cost-effective fabrication with design flexibility, potentially increasing the accessibility of this technology for widespread adoption. Future research should focus on optimizing these methods to integrate sustainable materials and enable the mass production of advanced 3D host designs.

#### 2.2.2. Mechanical Surface Modification Techniques

**Figure 4:**
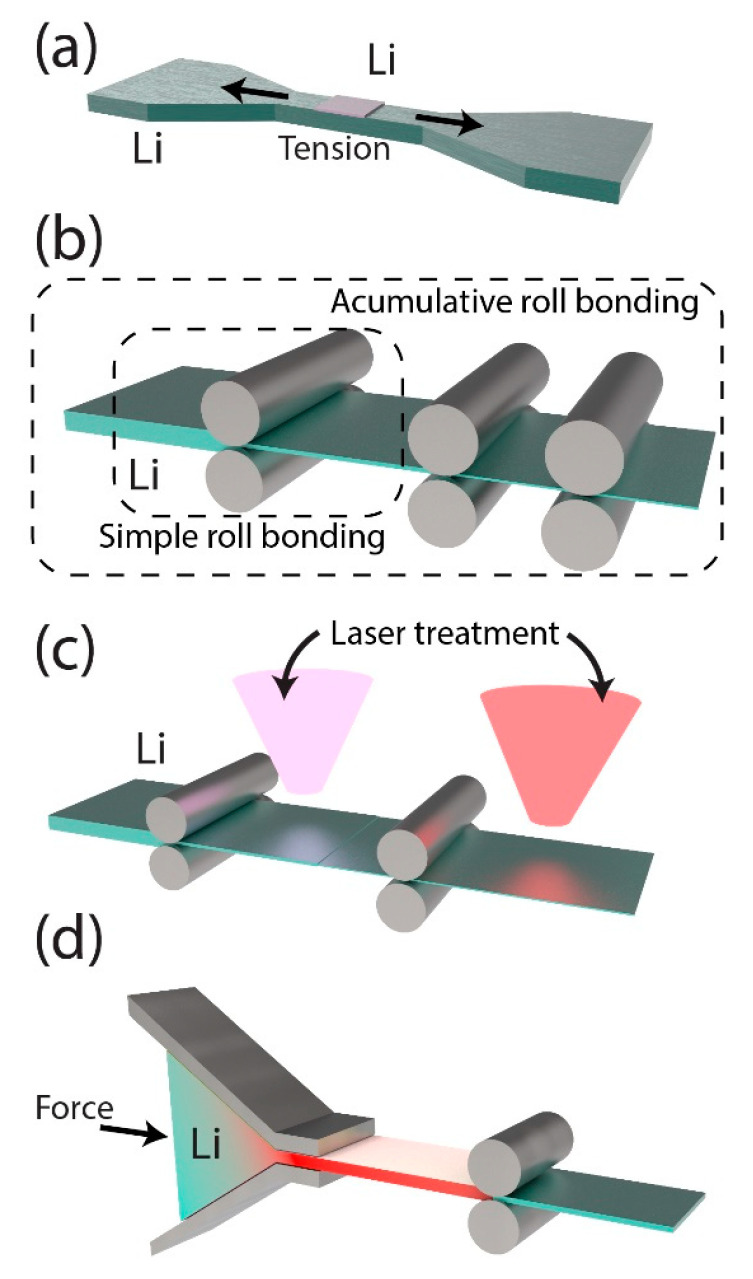
Mechanical surface pretreatment methods for lithium metal electrodes, redrawn based on previous studies: (**a**) a tension application aimed at improving surface uniformity and mitigating defects [52]; (**b**) roll bonding techniques designed to align lithium grains, refine microstructure, and enhance mechanical strength [53,54], (**c**) laser-assisted microstructuring to optimize surface roughness and chemical composition, reducing interfacial resistance [55]; and (**d**) extrusion combined with heat treatment to form a Li_2_CO_3_-enriched SEI, improving ionic conductivity and mechanical stability [56].

Mechanical surface modification provides an ex situ method for controlling the microstructure and performance of lithium metal negative electrodes, focusing on structural resilience and electrochemical stability. These techniques enhance lithium nucleation, suppress dendrite growth, and accommodate the volume fluctuations inherent to lithium cycling. By refining the surface properties and tailoring the microstructures (Figure 4), these strategies offer scalable and cost-effective solutions for improving long-term battery stability while addressing the challenges related to scalability, cost, and environmental impact.

One promising direction involves the creation of elastic and conductive hosts for lithium. For example, Xu et al. incorporated a 3D conductive polyurethane matrix (Figure 4a) [49]. This flexible structure adapts seamlessly to volumetric expansion and contraction, reducing mechanical stresses that would otherwise trigger dendrite formation. Figure 4a illustrates how the conductive framework supports a uniform lithium-ion flux, enabling consistent lithium deposition and stripping throughout cycling. However, the scalability of complex 3D designs is still a significant challenge. High production costs and material complexity must be addressed through cost-effective fabrication techniques that balance high performance and manufacturability.

In addition to elastic and conductive hosts, controlling the crystallographic orientation of lithium offers a compelling strategy for improving structural stability and lithium-ion transport. Hu et al. emphasized the importance of maintaining the {110} texture of lithium during a simple rolling process (Figure 4b) [50]. By aligning the lithium grains, they achieved uniform lithium deposition, thereby enhancing structural stability and cycling performance. However, this method introduces operational inefficiencies because periodic low-rate healing cycles are required to maintain electrode integrity. To address these limitations, Tan et al. developed the accumulative roll bonding (ARB) method, as shown in Figure 4b [51]. ARB refines the microstructure of lithium by repeatedly rolling and stacking, thereby reducing the grain size and improving the mechanical strength. This precise control of the microstructure suppresses dendrite formation and accommodates volume fluctuations, thereby bridging the gap between laboratory advancements and industrial applications. By enabling efficient production processes while maintaining performance, ARB presents a promising strategy for large-scale production.

Building on crystallographic alignment, advanced microstructuring techniques such as laser ablation open additional avenues for optimizing surface properties. For example, Kriegler et al. employed laser ablation to microstructure the lithium surface, optimizing both the surface roughness and chemical composition to improve lithium nucleation and reduce the interfacial resistance (Figure 4c) [52]. This technique not only removes contaminants and passivation layers but also enhances electrode–electrolyte interfaces, thereby facilitating ionic transport. Laser technology allows for precise surface modifications through parameter adjustments, thereby allowing for seamless integration into existing production lines. The combination of microstructuring precision and scalability highlights laser ablation as a strong candidate for industrial-scale applications.

Taking mechanical modifications a step further, heat treatment offers the opportunity to fine-tune surface properties and SEI characteristics. Nogales et al. explored the use of heat treatment to optimize both the chemical composition of a native oxide layer and the SEI (Figure 4d) [53]. By adjusting the extrusion speed and heat treatment temperature, a stable SEI enriched with Li_2_CO_3_ was obtained, enhancing ionic conductivity and mechanical robustness. This Li_2_CO_3_-enriched SEI minimizes side reactions while facilitating efficient ion transport, thereby extending the battery lifespan. However, maintaining consistent processing conditions across different battery systems is challenging. Variations in the temperature or extrusion parameters can compromise the SEI properties, highlighting the critical need for precise control over the manufacturing process.

These mechanical surface modification techniques illustrate how tailoring microstructures and surface properties can dramatically enhance the performance of lithium metal negative electrodes. Whether through elastic hosts, crystallographic alignment, or surface microstructuring, these strategies share the common goals of enhancing lithium nucleation, suppressing dendrite growth, and ensuring structural resilience. However, as these innovations advance, challenges related to scalability, reproducibility, and environmental sustainability remain key obstacles. Addressing these challenges is critical for translating laboratory successes into commercially viable solutions for next-generation lithium batteries.

### 2.3. Chemical and Electrochemical Surface Modification

Chemical and electrochemical surface modifications have significantly advanced the control of the reactivity of lithium metal negative electrodes by forming protective layers as ex situ pretreatments before assembly. These methods utilize liquid-phase compounds, electrolyte additives, and other reactive materials to suppress dendrite growth and improve interfacial properties. By carefully optimizing the reaction conditions and selecting tailored materials, researchers enhance both the chemical stability and ionic conductivity of lithium metal negative electrodes.

#### 2.3.1. Liquid-Phase Chemical Reactions for SEI Formation

**Figure 5:**
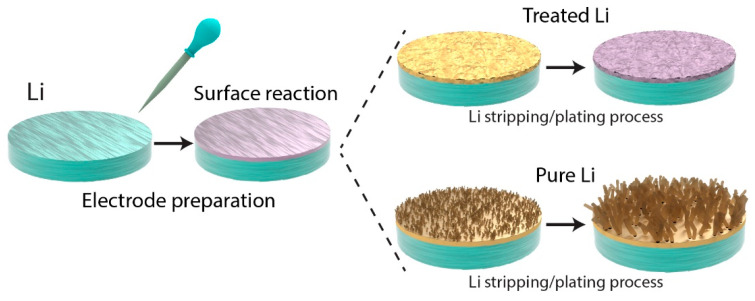
Surface reactions during liquid-phase chemical pretreatment for lithium electrodes, redrawn based on previous studies: treated Li exhibits a uniform and compact surface, enhancing lithium-ion diffusion and reducing dendrite growth during the stripping/plating process, whereas pure Li without pretreatment demonstrates uneven lithium deposition and significant dendrite formation [54,55,56,57,58,59,60,61,62].

Liquid-phase chemical reactions provide a practical and scalable pathway for enhancing the SEI in lithium metal negative electrodes and creating stable interphases that adapt to surface irregularities and mitigate side reactions (Figure 5). These reactions produce protective layers that reduce mechanical degradation and optimize lithium-ion transport. For example, Tao Liu et al. demonstrated how tin (II) iodide (SnI_2_) forms a protective barrier through a simple drop-coating method. This method yields a Li–Sn alloy and a lithium iodide (LiI) layer on the lithium surface, each contributing distinct benefits to SEI stability [54]. The Li–Sn alloy promotes uniform lithium deposition by providing a thiophilic interface, whereas LiI passivates the surface, effectively inhibiting dendrite growth. Despite these advantages, practical challenges such as the moisture sensitivity of SnI_2_ and its associated handling costs hinder its scalability. To address these challenges, researchers have explored alternative chemical formulations and structural modifications to improve SEI stability and scalability.

Zou et al. introduced a sulfamate-based SEI by immersing lithium in sulfanilic acid dissolved in dimethyl sulfoxide [55]. This SEI reduces surface diffusion barriers, ensuring long-term electrode stability and enhancing uniform lithium deposition. However, interactions between the sulfamate layer and bulk lithium during extended cycling degrade performance. This highlights the need for chemical formulations that balance initial performance and long-term reliability while ensuring durability without sacrificing ionic conductivity.

Building on these advancements, researchers have combined chemical and physical modifications to further improve SEI stability. Yang et al. used trimethoxy silane (TFOS) to create SEI coatings enriched with LiF, which provides chemical stability and passivates the electrode surface, thereby reducing undesirable reactions with the electrolyte [56]. Similarly, Lei Tan et al. investigated LiCl coatings that lower the Li^+^ diffusion energy barrier and facilitate smooth ion transport at the negative electrode interface [57]. The complementary strengths of these approaches, i.e., TFOS for electrochemical robustness and LiCl for enhanced ionic transport, highlight the potential of hybrid strategies that address distinct aspects of SEI stability. However, scalability, internal resistance, and mechanical stress remain key challenges, highlighting the need for materials that balance protective capacity and flexibility.

Further advancing these approaches, Krauskopf et al. incorporated phosphorus and nitrogen into an SEI using tris(N,N-tetramethylene)phosphoric acid triamide [58]. This modification enhances ionic conductivity and capacity retention, particularly in Li–S batteries, by fine-tuning the SEI composition to achieve efficient ion transport and long-term cycling stability. Similarly, Xie et al. engineered halide-rich SEIs containing lithium halides (LiF, LiBr, LiCl) through ex situ chemical treatments [59,60,61]. Among these, LiF stands out for its ability to foster stable ionic transport across the SEI and suppress dendrite growth [62]. Nevertheless, challenges such as long-term structural degradation and material depletion highlight the need for innovative materials and advanced coating techniques to maintain performance under extended cycling conditions.

As illustrated in Figure 5, a comparison between the lithium surfaces treated using liquid-phase reactions and the untreated lithium surface highlights the effectiveness of chemical pretreatments. The treated lithium surfaces exhibit superior dendrite suppression and enhanced lithium-ion transport during stripping and plating. These results highlight how liquid-phase chemical reactions that employ compounds such as SnI_2_, TFOS, or LiCl are advancing lithium metal battery technology while addressing the challenges related to material degradation and chemical interactions.

#### 2.3.2. Solvent Engineering for SEI Optimization

In addition to reactive coatings, optimizing solvent compositions has emerged as an effective ex situ strategy for stabilizing SEIs. Conventional electrolyte systems for lithium metal batteries are typically divided into ester-based and ether-based solvents. Ester-based electrolytes (e.g., ethylene carbonate, EC; ethyl methyl carbonate, EMC) offer high oxidative stability and compatibility with high-voltage cathodes, but they often form unstable SEIs on lithium. In contrast, ether-based solvents (e.g., 1,3-dioxolane, dimethoxyethane) promote more stable interfaces with lithium metal, although they are less stable at high voltages. This multifaceted influence highlights the need for tailored solvent formulations to meet diverse operating conditions and performance requirements.

Nogales et al. investigated the impact of varying the ethylene carbonate and ethyl methyl carbonate ratios on SEI properties and demonstrated that the solvent mixtures directly influence SEI ionic conductivity and stability [63]. Their findings emphasize the importance of refining solvent systems for optimizing the SEI composition and improving battery performance. Complementing these experimental studies, Fasulo et al. used computational modeling to elucidate the early steps in vinylene carbonate decomposition pathways on lithium surfaces, offering insights into SEI formation mechanisms [64]. The experimental validation of these predictions is essential to bridge the gap between theory and practical application.

Expanding on these efforts, Tao et al. developed an all-ester-based ternary electrolyte suitable for low-temperature cycling conditions [65]. This electrolyte enhances ionic conductivity and capacity retention at subzero temperatures by optimizing the solvation structure of lithium ions. However, broader adoption is hindered by challenges such as methyl acetate instability and the high cost of fluorinated solvents, highlighting the need for cost-effective alternatives. Beyond low-temperature solvents, the incorporation of specific additives such as LiNO_3_ demonstrates the potential of stabilizing SEIs under diverse operating conditions.

The importance of LiNO_3_-based strategies has been extensively demonstrated. Wen et al. demonstrated that a dual-salt electrolyte containing lithium trifluoroacetate (LiTFA) and LiNO_3_ improved SEI moisture tolerance and high-voltage performance by reinforcing the protective layer [66]. Similarly, Zhang et al. combined nitrate (NO^3−^) anions with bis(fluorosulfonyl)imide (FSI^−^) to form SEIs enriched with LiF and Li_3_N, enhancing both their mechanical strength and ionic transport [67]. These findings highlight the versatility of LiNO_3_ as a key additive while emphasizing the necessity of a precise formulation to minimize the side reactions associated with NO^3−^ reactivity.

**Figure 6:**
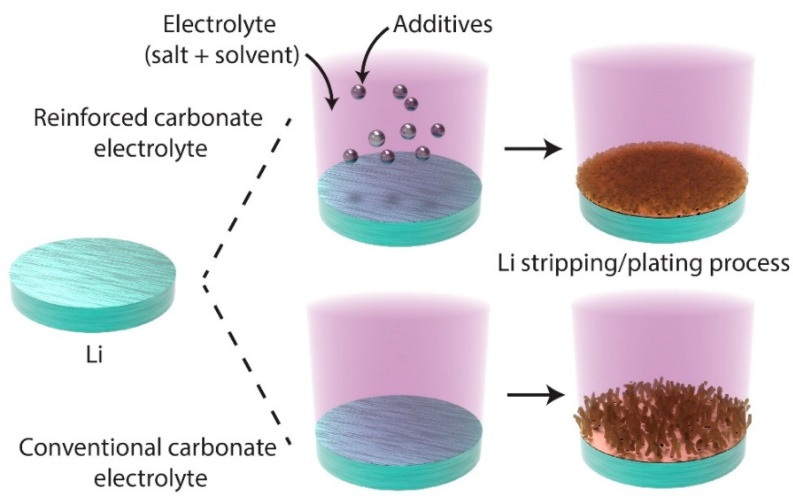
Comparison of lithium stripping/plating behavior between reinforced carbonate electrolytes containing salt additives, which enhance deposition uniformity and suppress dendrite growth, and conventional carbonate electrolytes, which lead to uneven lithium growth and dendrite formation. Redrawn based on previous studies [63,64,65,66,67,68].

Park et al. extended these applications by integrating LiNO_3_ into 3D host structures, illustrating its potential for enhancing battery performance in hybrid designs. Figure 6 shows the combined effects of LiNO_3_ and 3D hosts, which promote the formation of an inorganic-rich SEI layer, thereby improving stability and efficiency [68]. This integration demonstrates how advanced structural designs can complement chemical additives to address the multifaceted challenges associated with lithium negative electrodes.

Figure 6 compares the performance of reinforced carbonate electrolytes containing salt additives and conventional electrolytes. The reinforced systems significantly outperformed their conventional counterparts, exhibiting superior stability and efficiency during lithium stripping and plating. Additionally, localized high-concentration electrolytes (LHCEs) have emerged as a promising strategy to stabilize lithium metal interfaces. By tailoring the solvation structure and reducing the activity of free solvents, LHCEs enable the formation of inorganic-rich SEIs that suppress dendrite growth and improve coulombic efficiency. Although not the primary focus of this review, their relevance highlights the growing emphasis on electrolyte design as a complementary approach to surface pretreatments. While these solvent and additive strategies often involve in situ SEI formation, they are discussed here due to their relevance as part of preconditioning treatments that can complement ex situ approaches. Their integration supports the stabilization of lithium interfaces when combined with surface-engineered anodes. Finally, although anode-free lithium metal batteries are outside the scope of this review, we recognize their growing significance. Insights from lithium surface engineering such as SEI stabilization and dendrite suppression may offer valuable contributions to these emerging systems.

## 3. Conclusions and Perspectives

### 3.1. Conclusions

Advances in ex situ and in situ surface treatments for lithium metal negative electrodes have significantly mitigated the challenges hindering the commercialization of lithium metal batteries. Key developments include SEI formation techniques, polymer-based protective layers, structural modifications, and chemical surface modifications, all of which improve the performance and stability of lithium metal electrodes. The evolution from simple to complex multicomponent coatings highlights the progress toward a balance between mechanical strength and ionic conductivity. Single-component coatings, such as LiF, provide excellent chemical stability, whereas multicomponent SEIs containing compounds such as Li_2_S and Li_2_SO_3_ enhance ionic transport and ensure uniform lithium deposition.

Ex situ polymeric layers provide elasticity and self-healing properties, effectively suppressing dendrite growth and adapting to structural changes during cycling. Although these advances show significant potential, challenges related to long-term stability and scalability in manufacturing should be addressed to facilitate commercial adoption. Similarly, ex situ structural modifications, such as 3D host structures made from NiCo nanocubes and carbonized bacterial cellulose nanofibers, improve lithium-ion transport and minimize dendrite formation. Despite these benefits, the reliance on complex fabrication processes and expensive raw materials remains a barrier to large-scale production. In addition, mechanical surface modifications, such as ARB and laser ablation, can refine the microstructure and enhance nucleation and electrode resilience. These techniques provide valuable insights into tailoring the properties of lithium metal surfaces, thereby extending battery life and improving cycling performance. To illustrate these advancements with greater clarity, Table 1 compares the electrochemical performance metrics, such as coulombic efficiency, capacity retention, and testing conditions, of various techniques. This comparative analysis offers valuable insights into optimizing the performance of lithium electrodes under diverse experimental conditions.

**Figure 7:**
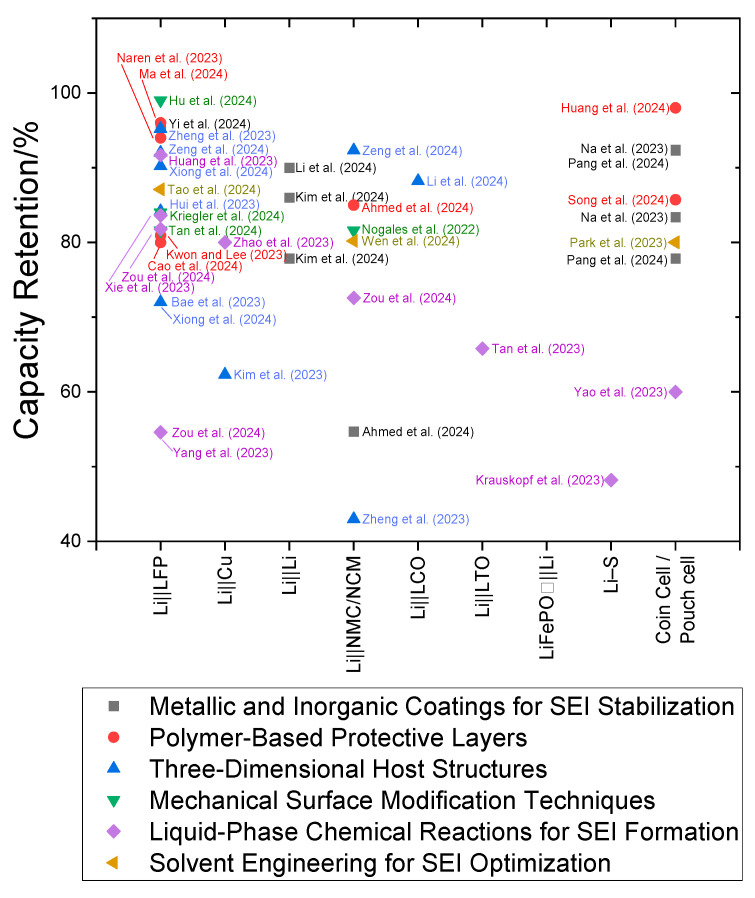
The capacity retention performance of various lithium-based cells subjected to different ex situ surface treatment strategies. The *x*-axis categorizes the data by battery type (e.g., Li||LFP, Li||Cu, Li–S), enabling clearer distinction between different cell configurations. The references cited in the figure correspond to the studies summarized in Table 1, facilitating a direct comparison between retention behavior and treatment method across distinct electrochemical systems [19,20,21,22,24,25,29,31,32,33,34,35,36,40,41,42,43,44,45,46,47,50,51,52,53,55,56,57,58,59,60,61,62,65,66,68].

Table 1 and Figure 7 provide a comprehensive comparison of various ex situ surface treatments in terms of their effects on pivotal battery performance metrics. Table 1 presents numerical metrics, such as coulombic efficiency and cycle retention, and Figure 7 highlights the variability in capacity retention across different techniques under varying experimental conditions. Notably, the metallic and inorganic coatings (black squares) cluster near 100% capacity retention, highlighting their effectiveness in stabilizing the SEI layer and enhancing cycling stability. In contrast, polymer-based protective layers (red circles) and solvent engineering techniques (yellow rotated triangles) exhibit broader variability. Polymer layers exhibit significant variability (~60–90%), possibly due to variations in the polymer structure and electrolyte compatibility, whereas solvent engineering techniques exhibit consistent retention (~70%), reflecting better SEI formation control. Although liquid-phase chemical reactions (purple diamonds) exhibit moderate variability, they typically achieve retention values of ~75–80%), indicating their controlled SEI formation. Mechanical surface modification techniques (the inverted green triangles) consistently exhibit a high capacity retention of approximately 90%, indicating their potential for reliable long-term cycling stability. In addition, 3D host structures (blue triangles) demonstrate a wide range of retention values ~60–85%, highlighting their promise but also the need for further optimization in design and synthesis.

Metallic coatings, such as Li||LiHg film cells, exhibit near-perfect coulombic efficiency (~95.4%) and capacity retention (124 mAh g^−1^ over 200 cycles), consistently outperforming other techniques. In contrast, polymer-based protective layers exhibit a notable ability to suppress dendrite growth, as observed in STCPL@Li||NMC811, which maintains ~85% capacity after 300 cycles at a C/2 charge–discharge rate. Although 3D host structures, such as ZnNNi_3_@CC||Li, exhibit capacity retention of ~85% over 200 cycles, their comparatively lower performance suggests the need for scalable and cost-effective designs. Future research should explore automated manufacturing processes and template-free synthesis methods to enhance the scalability of 3D host structures. Mechanical surface modification techniques, such as ARB, exhibit near-perfect coulombic efficiency and negligible capacity loss. However, their scalability and industrial adoption are hindered by the need for further process optimization.

Despite these promising results, Figure 7 highlights substantial variability in performance across techniques, largely due to differences in experimental setups, such as temperature, current density, and electrolyte composition. Standardizing testing protocols for key parameters, such as temperature, current density, and electrolyte composition, will ensure reproducibility across studies, foster collaborations among researchers, and accelerate the industrial adoption of advanced battery technologies.

### 3.2. Critical Perspectives

Although significant progress has been achieved, transitioning these advances from research to commercial-scale applications remains hindered by persistent challenges. Importantly, scalability continues to be a major obstacle, particularly for 3D host structures and other ex situ structural modifications, because their fabrication processes typically depend on expensive, scarce materials and complex manufacturing techniques. Although mechanical surface modifications are promising, they require further optimization to balance scalability, cost-effectiveness, and performance.

In addition to scalability, uniformity of the ex situ surface treatments presents a critical challenge for practical implementation. While many approaches achieve excellent results at the laboratory scale, reproducing uniform coatings across large lithium surfaces remains difficult. Non-uniform SEI layers can lead to inhomogeneous lithium-ion flux, localized dendrite formation, and reduced long-term cycling stability. Techniques such as vapor-phase deposition, dip coating, and thermal treatment must be further refined to ensure consistent layer thickness, chemical composition, and interfacial stability across different electrode sizes and geometries. Addressing these issues is crucial for achieving reliable and scalable integration of ex situ strategies in industrial battery manufacturing.

Although most current ex situ strategies are evaluated under generalized conditions, future efforts should consider cathode-specific interactions. For instance, sulfur-based cathodes release soluble polysulfides, oxide cathodes such as NMC, LCO, or NCA can release transition metal ions, and high-voltage cathodes may promote electrolyte oxidation each presenting unique challenges at the lithium interface. Tailoring surface treatments to account for these chemistries may lead to improved compatibility and cycling stability in full-cell systems.

In addition, chemical and electrochemical surface modifications, including ex situ liquid-phase reactions and electrolyte engineering, have demonstrated their potential for SEI stabilization through the use of reactive compounds and additives such as LiNO_3_, KI, and brominated compounds. However, despite their promise, these strategies typically rely on hazardous or costly chemicals, raising substantial environmental and safety concerns. For example, although brominated compounds effectively promote uniform lithium deposition, they are associated with significant toxicity, limiting their practical application. Fluorinated compounds, known for their chemical stability, can reduce toxicity even though cost and scalability are associated challenges, whereas organic additives offer another promising route for safer SEI stabilization. Addressing these limitations requires a holistic approach that integrates structural innovations with chemical strategies, ensuring that advances in one domain do not worsen challenges in another.

Another important limitation when reviewing ex situ surface treatments is the lack of standardized testing conditions across the literature. Variations in current density, electrolyte composition, cell format, temperature, and cycling protocols make direct performance comparisons difficult. While Table 1 summarizes representative results, caution should be taken when drawing quantitative conclusions from these metrics. Future studies should aim to adopt unified testing protocols to facilitate reproducibility and fair benchmarking across different strategies.

An additional, often overlooked factor is the preparation method of the lithium metal substrate itself. Lithium surfaces fabricated by mechanical pressing, electrochemical deposition, or slurry coating can differ substantially in terms of surface roughness, grain orientation, and especially the composition and thickness of their native oxide layers. These intrinsic differences affect lithium nucleation behavior and the chemical interactions involved in SEI formation. As such, the effectiveness of any surface treatment may strongly depend on the initial state of the lithium. Future studies should systematically investigate how substrate fabrication history influences SEI development to better compare and standardize ex situ strategies. Initial lithium foil thickness, which determines the available lithium reservoir and overcapacity margin, is a key variable that can strongly influence cycle life outcomes. However, this parameter remains inconsistently reported across the literature, limiting the ability to normalize and compare the effectiveness of ex situ surface treatments.

To overcome these barriers, future research should focus on cost-effective manufacturing techniques and environmentally benign additives, thereby reducing the reliance on hazardous or expensive chemicals. This can include exploring renewable or abundant material sources, such as biopolymers or naturally occurring compounds such as cellulose or chitosan, optimizing synthesis processes for higher efficiency, and leveraging advanced computational tools to predict and design safer additive chemistries. For example, machine learning models can be used to identify optimal combinations of additives for enhanced SEI performance while minimizing environmental impacts. By integrating scalable processes with innovative material designs, researchers can address existing limitations and support the transition of ex situ surface treatments from laboratory studies to commercial applications. Ultimately, these efforts could pave the way for the large-scale deployment of greener lithium-ion batteries in electric vehicles and grid storage systems, significantly reducing their environmental footprint while enhancing their commercial viability. Finally, we note that while many of the reviewed strategies demonstrate promising electrochemical performance, aspects such as process scalability and cost-efficiency remain underexplored in the current literature. Future research should address these techno-economic factors to support practical implementation. It is also worth noting that recent advances in separator modification and solid-state electrolyte development, such as those reported by Sun et al., Peng et al., and Li et al. [69,70,71], have provided valuable insights into lithium interface stabilization and dendrite suppression. While these strategies do not directly involve ex situ surface treatments of lithium metal, they represent complementary interface strategies and contribute meaningfully to the broader field of interfacial engineering. Moreover, they may inspire hybrid approaches that integrate both ex situ and in situ elements to enhance lithium metal battery performance.

### 3.3. Future Directions

To realize the full potential of lithium metal negative electrodes in next-generation batteries, future research should prioritize the following:

- Material Innovation: Developing cost-effective, abundant, and environmentally friendly materials is critical for advancing lithium-based technologies. Exploring alternatives such as aluminum- and silicon-based hosts and bio-derived polymers such as cellulose and chitosan can significantly reduce costs and mitigate environmental impacts. For example, bio-derived polymers exhibit compatibility with scalable processes and improved recyclability, making them promising candidates for future applications. Material innovation should also focus on enhancing compatibility with scalable manufacturing processes and improving recyclability, thereby enabling a more circular economy for battery materials.
- Scalable Manufacturing Processes: Building upon material innovation, scalable manufacturing processes will play a pivotal role in enabling commercial-scale production. Advances in fabrication technologies, including additive manufacturing and roll-to-roll processing, enable the large-scale production of complex structures and coatings. Techniques such as 3D host structures or polymeric protective layers may benefit from scalable processes such as roll-to-roll or continuous coating technologies for industrial production. Emphasis should be placed on reducing process costs and improving the throughput of high-precision techniques to meet commercial demands.
- Computational Modeling and Simulation: Leveraging computational tools to predict SEI formation mechanisms and optimize material properties can accelerate the design of effective surface treatments. Integrating machine learning with computational modeling may further enhance the efficiency and accuracy of experimental efforts and reduce development timelines. Computational models can also help design hybrid systems of surface treatments (such as polymer-based and metallic coatings) for optimal performance, thereby minimizing the trial-and-error phase in experimental setups. By combining these tools with experimental validation, researchers can establish a robust framework for designing next-generation surface treatments.
- Standardization and Long-Term Testing: Establishing standardized testing protocols is essential for enabling consistent comparisons across different technologies and providing insights into practical applications. Long-term cycling tests under realistic operating conditions, such as variable temperatures and current densities, are necessary to evaluate durability and ensure reliable performance. Adopting standardized protocols for capacity retention and Coulombic efficiency measurements, as shown in the comparative table, is important for reliably assessing the viability of various surface treatments across diverse lithium metal electrode configurations.
- Environmental Sustainability: Future research should incorporate comprehensive lifecycle assessments to evaluate and minimize the environmental footprint of battery production. For example, strategies using CO_2_-pretreated lithium or environmentally friendly polymer layers can significantly reduce the ecological impact. This approach not only reduces CO_2_ emissions but also enhances the reactivity and deposition uniformity of lithium. Such innovations will be essential for improving performance and sustainability, aligning with global sustainability goals such as net-zero carbon targets.

By collectively addressing these priorities, future research can bridge the gap between laboratory-scale advances and commercial applications, thereby accelerating the adoption of lithium metal electrodes in next-generation batteries. A multidisciplinary approach that integrates material science, engineering, computational tools, and environmental assessments will be essential for achieving this goal, paving the way for sustainable and scalable lithium metal technologies.

## Figures and Tables

**Table 1:** Summary of ex situ surface treatments for lithium and their performance metrics.

Cell Type	Coulombic Efficiency	Capacity Retention	Ref.
Metallic and Inorganic Coatings for SEI Stabilization			
Bare Li||LFP full cell	Not specified	28% retention after 200 cycles (capacity decreased to 35 mAh g^−1^)	[19]
LCZO||LFP full cell	Not specified	95.4% retention after 200 cycles (capacity of 124 mAh g^−1^)	[19]
Li||LiHg film cell	100% over 160 cycles	Over 90% retention ~100 mAh g^−1^ after 100 cycles at 2 C rate	[20]
Li||LCO coin cell	99.2% after 900 cycles	Theoretical capacity 145 mA g^−1^ 125 mAh g^−1^ discharge capacity after 40 cycles at 120 °C heat treatment. 86% retention approximately	[24]
DFFSA-Li coin cell and pouch cell	Not specified	77.85% retention after 1200 cycles in Li LCO coin cell at 1C discharging (from 153.98 mAh g^−1^ to 119.88 mAh g^−1^). 92.3% retention after 90 cycles in pouch cell with NCM811. 84.7% retention after 100 cycles in pouch cell with NCM811. 83.1% retention after 110 cycles in pouch cell with 100 µm DFFSA-Li. 86.67 mAh g^−1^ after 5 C charge/discharge.	[22]
Bare Li coin cell and pouch cell	Not specified	28% retention after 530 cycles in Li LCO coin cell (from 145.7 mAh g^−1^ to 79.7 mAh g^−1^). 23 mAh g^−1^ after 65 cycles in pouch cell.	[22]
CO₂ pre-treated Li coin cell	99.2% after 1200 cycles	125 mAh g^−1^ initial discharge capacity 77.85% retention after 1200 cycles.	[25]
Bare Li coin cell	Not specified	145.7 mAh g^−1^ initial discharge capacity 54.7% retention, 79.7 mAh g^−1^ after 530 cycles.	[25]
Li||SMGF@NF half cell	99.2% Stable for 300 cycles at 0.5 C and 500 cycles at 1 C	92.4% retention 113.4 mAh g^−1^ after 1000 cycles at 5 C.	[21]
Li||MGF@NF half cell	Not specified	Sharp decay after 246 cycles at 5 C. At 0.5 C, the capacity was 146.8 mAh g^−1^,	[21]
Li||NF half cell	Not specified	Sharp decay after 164 cycles at 5 C. At 0.5 C, the capacity retention was 83.4% of the initial capacity.	[21]
Polymer-Based Protective Layers			
Li||LiFePO₄ (LFP) full cell	Not specified	Initial capacity was 143.2 mAh g^−1^, with 106.3 mAh g^−1^ remaining after 350 cycles. 80.94% retention after 800 cycles.	[31]
Li||LiFePO₄ (LFP) full cell with P(St-MaI)@Li anode	Not specified	Initial capacity: 155 mAh g^−1^ at 1 C 96% retention, After 930 cycles: (around 148 mAh g^−1^)	[33]
Li||LiFePO₄ (LFP) full cell	99.8% during 250 cycles at a low N/P ratio (~3)	First cycle capacity: 148.4 mAh g^−1^ (at 1 C rate) 85.7% retention. after 500 cycles 145 mAh g^−1^	[34]
LiFePO₄||Li (SP-lithium)	Not specified	Stable discharge capacity of 140 mAh g^−1^ after 600 cycles 98% retention after 300 cycles.	[36]
LiFePO₄ full cell with P-PTh-Li	Not specified	Initial reversible capacity: 146.8 mAh g^−1^ at 1 C. After 500 cycles, the capacity retention is 94.0%, with a capacity of 138.0 mAh g^−1^	[32]
STCPL@Li||NMC811	Not specified	1st cycle is mentioned as 192.5 mAh g^−1^ 85% retention, At C/2 charge/discharge rate, after 300 cycles	[29]
Li||NMC811	Not specified	1st cycle is mentioned as 193.6 mAh g^−1^ 24% retention, After 300 cycles.	[29]
C-Li@P	Not specified	Initial capacity (774 mAh g^−1^) retaining > 80% capacity after 200 cycles.	[35]
Three-Dimensional Host Structures			
CBC-Li||LFP	Not specified	Initial capacity 155 mAh g^−1^ 90.2% retention after 700 cycles	[41]
Pure Li||LFP	Not specified	Initial capacity 155 mAh g^−1^ 62.3% retention after 700 cycles	[41]
Li||LFP	Not specified	151.74 mAh g^−1^ in the first cycle 95.2% retention after 400 cycles	[42]
Li/CC-Ag||LiFePO₄ full cell	Approaching 100% for 300 cycles at 1 C	92% retention after 250 cycles (from 148 to 136 mAh g^−1^)	[44]
LFP||LAD-SSC@CF@Li full cell	Not specified	149.2 mAh g^−1^ in the initial cycle 88.2% capacity retention after 300 cycles at 1 C	[45]
Li||NCM811	Not specified	Cell type: Batteries deliver 179.07 mAh g^−1^ 84.16% retention after 100 cycles.	[43]
Li/CC-Ag||NCM622 full cell	Not specified	92.3% retention after 200 cycles (from 171.2 to 162.9 mAh g^−1^)	[44]
Li||LCO pouch cell with NSNF-hosted L	Not specified	176.5 mAh g^−1^ after 8 cycles 72% retention, 127.5 mAh g^−1^ after 200 cycles	[46]
ZnNNi_3_@CC||Li	High efficiency (>99%) across long cycles	Initial capacity of 143.4 mAh g^−1^ 72% retention after 1500 cycles.	[40]
10Au@2D-Cu	Not specified	First discharge capacity is 159 mAh g^−1^ 5% retention, 8 mAh g^−1^ in the 100th cycle.	[42]
20Au@2D-Cu	Not specified	Exhibits 160 mAh g^−1^ in the 1st cycle 18.75% retention 30 mAh g^−1^ in the 100th cycle.	[42]
20Au@3D-Cu:	Not specified	Exhibits 162 mAh g^−1^ in the 1st cycle 43% retention, 70 mAh g^−1^ in the 100th cycle.	[42]
Mechanical Surface Modification Techniques			
Li||LFP full cell	Not specified	Initial discharge capacity over 150 mAh g^−1^ 81.6% retention after 500 cycles at 5.0 C.	[51]
Li||LiFePO₄ (LFP) full cell	Near 100%	80 mAh g^−1^ during 300 cycles at 5 C 99% retention	[50]
Li||NCM+LMO coin cell	Not specified	Initial discharge capacity over 112 mAh g^−1^ 84% retention after 60 cycles and 53% capacity retention after 60 cycles in non-treated sample.	[52]
ELMA-based pouch cells	Energy density (375 Wh/kg) and maintained stability over 200 cycles	Not specified	[49]
Liquid-Phase Chemical Reactions for SEI Formation			
Li–SA@Li||LFP full cell	Not specified	Initial discharge capacity of 157.7 mAh g^−1^ at 0.5 C 81.8% retention after 200 cycles	[55]
Bare Li||LFP full cell	Not specified	Initial capacity of 141.6 mAh g^−1^ 54.6% retention	[55]
TFOS-Li||LFP full cell	99.92% after 1650 cycles	Initial capacity ~250 mAh g^−1^ at 1.5 C Capacity retention: Not specified	[56]
Cu-mesh@Ag-Li||LFP	over > 99.7%	Initial discharge capacities 167.1 mAh g^−1^ 91.7% retention of initial capacity after 800 cycles at 2 C.	[61]
Li||Cu cells (BTFM-based electrolytes)	99.72% over 500 cycles	80% retention over 600 cycles	[62]
Li–SA@Li||NCM full cell	Not specified	72.6% retention after 200 cycles at 0.5 C	[55]
LiCl@Li||LTO full cell	Not specified	Initial capacity of 155 mAh g^−1^ 65.8% retention after 1000 cycles	[57]
Li-S full cell	Not specified	48.2% retention capacity retention after 120 cycles	[58]
LFP-Sn||LiI@Li	99.8% after 1000 cycles	120.6 mAh g^−1^ initial capacity Capacity retention: Not specified	[54]
LFP||LFCB811@Li full cell	99.9%	Cell type with initial capacity 127.4 mAh g^−1^ 83.6% retention after 1000 cycles	[59]
LiBr@Li full cell	99.9%	Initial capacity ~155 mAh g^−1^ 60% retention after 500 cycles	[60]
Solvent Engineering for SEI Optimization			
Li||LFP cells (F/MA electrolyte)	97% for 200 cycles	87.1% retention after 400 cycles	[65]
Li||Cu cells with DAE1:1, LiNO_3_:LiFPFSI	97.2% at the 160 cycle	Not specified	[67]
Li||NCM523 cells with LTFAN	Not specified	80.2% retention capacity retention after 300 cycles at 1 C	[66]
3D-CNS1600@LiNO_3_	95.3–98%	over 80% retention after 200 cycles	[68]

## Data Availability

No new data were created or analyzed in this study.

## References

[1] Xu W., Wang J., Ding F., Chen X., Nasybulin E., Zhang Y., Zhang J.G. (2014). Lithium Metal Anodes for Rechargeable Batteries. Energy Environ. Sci..

[2] Cheng X.-B., Zhang R., Zhao C.-Z., Zhang Q. (2017). Toward Safe Lithium Metal Anode in Rechargeable Batteries: A Review. Chem. Rev..

[3] Wu H., Wu Q., Chu F., Hu J., Cui Y., Yin C., Li C. (2019). Sericin Protein as a Conformal Protective Layer to Enable Air-Endurable Li Metal Anodes and High-Rate Li-S Batteries. J. Power Sources.

[4] Li C., Wei J., Li P., Tang W., Feng W., Liu J., Wang Y., Xia Y. (2019). A Dendrite-Free Li Plating Host towards High Utilization of Li Metal Anode in Li–O_2_ Battery. Sci. Bull..

[5] Zu C., Azimi N., Zhang Z., Manthiram A. (2015). Insight into Lithium–Metal Anodes in Lithium–Sulfur Batteries with a Fluorinated Ether Electrolyte. J. Mater. Chem. A.

[6] Li T., Zhang X.Q., Shi P., Zhang Q. (2019). Fluorinated Solid-Electrolyte Interphase in High-Voltage Lithium Metal Batteries. Joule.

[7] Jagger B., Pasta M. (2023). Solid Electrolyte Interphases in Lithium Metal Batteries. Joule.

[8] Liu W., Liu P., Mitlin D. (2020). Review of Emerging Concepts in SEI Analysis and Artificial SEI Membranes for Lithium, Sodium, and Potassium Metal Battery Anodes. Adv. Energy Mater..

[9] Ye L., Li X. (2021). A Dynamic Stability Design Strategy for Lithium Metal Solid State Batteries. Nature.

[10] Cheng X.B., Zhao C.Z., Yao Y.X., Liu H., Zhang Q. (2019). Recent Advances in Energy Chemistry between Solid-State Electrolyte and Safe Lithium-Metal Anodes. Chem.

[11] Chen S., Zheng J., Mei D., Han K.S., Engelhard M.H., Zhao W., Xu W., Liu J., Zhang J.-G., Chen S. (2018). High-Voltage Lithium-Metal Batteries Enabled by Localized High-Concentration Electrolytes. Adv. Mater..

[12] Yu Z., Cui Y., Bao Z. (2020). Design Principles of Artificial Solid Electrolyte Interphases for Lithium-Metal Anodes. Cell. Rep. Phys. Sci..

[13] Li N.-W., Yin Y.-X., Yang C.-P., Guo Y.-G. (2016). An Artificial Solid Electrolyte Interphase Layer for Stable Lithium Metal Anodes. Adv. Mater..

[14] Zhang R., Li N.W., Cheng X.B., Yin Y.X., Zhang Q., Guo Y.G. (2017). Advanced Micro/Nanostructures for Lithium Metal Anodes. Adv. Sci..

[15] Chen Y., Ke X., Cheng Y., Fan M., Wu W., Huang X., Liang Y., Zhong Y., Ao Z., Lai Y. (2020). Boosting the Electrochemical Performance of 3D Composite Lithium Metal Anodes through Synergistic Structure and Interface Engineering. Energy Storage Mater..

[16] Tan L., Sun Y., Wei C., Tao Y., Tian Y., An Y., Zhang Y., Xiong S., Feng J., Tan L. (2021). Design of Robust, Lithiophilic, and Flexible Inorganic-Polymer Protective Layer by Separator Engineering Enables Dendrite-Free Lithium Metal Batteries with LiNi_0.8_Mn_0.1_Co_0.1_O_2_ Cathode. Small.

[17] Shin W.-K., Kannan A.G., Kim D.-W. (2015). Effective Suppression of Dendritic Lithium Growth Using an Ultrathin Coating of Nitrogen and Sulfur Codoped Graphene Nanosheets on Polymer Separator for Lithium Metal Batteries. ACS Appl. Mater. Interfaces.

[18] Xiang J., Yuan L., Shen Y., Cheng Z., Yuan K., Guo Z., Zhang Y., Chen X., Huang Y., Xiang J. (2018). Improved Rechargeability of Lithium Metal Anode via Controlling Lithium-Ion Flux. Adv. Energy Mater..

[19] Yi L., Wang Z., Chen X., Xing J., Huang H., Wei C., Zhao Q., Zhou A., Li J. (2024). An Electron-Insulating Li_2_O Protection Layer Endowing a Li–Cu–Zn Ternary Alloy Composite Anode with High Performance. Chem. Commun..

[20] Li Q., Liu Y., Zhang Z., Chen J., Yang Z., Deng Q., Mumyatov A.V., Troshin P.A., He G., Hu N. (2024). Construction of Dynamic Alloy Interfaces for Uniform Li Deposition in Li-Metal Batteries. Energy Environ. Mater..

[21] Na Z., Li W., Wang X., Liu D., Sun J., Lang M., Liu W., Huang G. (2023). Mesoporous Gold Film with Surface Sulfur Modification to Enable Dendrite-Free Lithium Plating/Stripping for Long-Life Lithium Metal Anodes. Small Methods.

[22] Pang M., Jiang Z., Luo C., Yao Z., Fu T., Pan T., Guo Q., Li Y., Xiong S., Zheng C. (2024). A Surface Chemistry-Regulated Gradient Multi-Component Solid Electrolyte Interphase for a 460 W h Kg^−1^ Lithium Metal Pouch Cell. Energy Environ. Sci..

[23] Wang T., Liu X., Huang S., Lu J., Li J., Ge S., Wang C. (2024). Development of Polymer-Based Artificial Solid Electrolyte Interphase for Safer Li-Metal Batteries: Challenges, Strategies and Prospects. Nano Energy.

[24] Nogales P.M., Lee S., Yang S., Jeong S.K. (2024). Understanding the Impact of Li_2_CO_3_ Distribution within Solid Electrolyte Interphases on Lithium Metal via Thermal Conditioning. Electrochim. Acta.

[25] Kim Y., Maldonado Nogales P., Lee C., Jeong S.K. (2024). Enhancing Electrochemical Characteristics of Li-Metal Electrodes: The Impact of Pre-Treatment via CO_2_ Gas Reaction. ChemElectroChem.

[26] Schmitz R., Müller R., Krüger S., Schmitz R.W., Nowak S., Passerini S., Winter M., Schreiner C. (2012). Investigation of Lithium Carbide Contamination in Battery Grade Lithium Metal. J. Power Sources.

[27] Golozar M., Hovington P., Paolella A., Bessette S., Lagacé M., Bouchard P., Demers H., Gauvin R., Zaghib K. (2018). In Situ Scanning Electron Microscopy Detection of Carbide Nature of Dendrites in Li-Polymer Batteries. Nano Lett..

[28] Lin X., Shen Y., Yu Y., Huang Y. (2024). In Situ NMR Verification for Stacking Pressure-Induced Lithium Deposition and Dead Lithium in Anode-Free Lithium Metal Batteries. Adv. Energy Mater..

[29] Ahmed R.A., Koirala K.P., Zhao Q., Kim J.-M., Anderson C., Wang C., Zhang J.-G., Xu W. (2024). Surface-Treated Composite Polymer as a Stable Artificial Solid Electrolyte Interphase Layer for Lithium Metal Anode. ACS Appl. Energy Mater..

[30] Chen K., Guo X., Chen M. (2023). Controlled Radical Copolymerization toward Well-Defined Fluoropolymers. Angew. Chem.-Int. Ed..

[31] Liu Z., Zhang Q., Song X., Shi Y., Zhu X., Liu X., Zhou Y., Chen Z., Feng Y., Chen S. (2024). Construction of Inorganic/Polymer Tandem Layer on Li Metal with Long-Term Stability by LiNO_3_ Concentration Gradient Electrolyte. Small.

[32] Cao S., Ning J., He X., Wang T., Xu C., Chen M., Wang K., Zhou M., Jiang K. (2024). In Situ Plasma Polymerization of Self-Stabilized Polythiophene Enables Dendrite-Free Lithium Metal Anodes with Ultra-Long Cycle Life. Small.

[33] Naren T., Kuang G.C., Jiang R., Qing P., Yang H., Lin J., Chen Y., Wei W., Ji X., Chen L. (2023). Reactive Polymer as Artificial Solid Electrolyte Interface for Stable Lithium Metal Batteries. Angew. Chem.-Int. Ed..

[34] Ma C., Zou S., Wu Y., Yue K., Cai X., Wang Y., Nai J., Guo T., Tao X., Liu Y. (2024). A Triply-Periodic-Minimal-Surface Structured Interphase Based on Fluorinated Polymers Strengthening High-Energy Lithium Metal Batteries. Angew. Chem.-Int. Ed..

[35] Song H., Lee J., Sagong M., Jeon J., Han Y., Kim J., Jung H.G., Yu J.S., Lee J., Kim I.D. (2024). Overcoming Chemical and Mechanical Instabilities in Lithium Metal Anodes with Sustainable and Eco-Friendly Artificial SEI Layer. Adv. Mater..

[36] Kwon N.A., Lee J. (2023). Won Surface Modification of Lithium Metal Anode with Lithium Silicate-Lithium Phosphate Composite Layer for Enhanced Cycling Stability. Mater. Chem. Phys..

[37] Golozar M., Paolella A., Demers H., Bessette S., Lagacé M., Bouchard P., Guerfi A., Gauvin R., Zaghib K. (2019). In Situ Observation of Solid Electrolyte Interphase Evolution in a Lithium Metal Battery. Commun. Chem..

[38] Lang S., Colletta M., Krumov M.R., Seok J., Kourkoutis L.F., Wen R., Abruña H.D. (2023). Multidimensional Visualization of the Dynamic Evolution of Li Metal via In Situ/Operando Methods. Proc. Natl. Acad. Sci. USA.

[39] Zhang X.S., Wan J., Shen Z.Z., Lang S.Y., Xin S., Wen R., Guo Y.G., Wan L.J. (2024). In Situ Analysis of Interfacial Morphological and Chemical Evolution in All-Solid-State Lithium-Metal Batteries. Angew. Chem. Int. Ed..

[40] Huang L., Li W., Liang L., Zeng B., Lin X., Xie Y., Du L., Song H., Lu Y., Cui Z. (2024). Anti-Perovskite Nitrides as Chemically Stable Lithiophilic Materials for Highly Reversible Li Plating/Stripping. Energy Storage Mater..

[41] Xiong G., Yu J., Xing Y., Yang P., Zhang S. (2024). Intrinsic Lithiophilic Carbon Host Derived from Bacterial Cellulose Nanofiber for Dendrite-Free and Long-Life Lithium Metal Anode. Nano Res..

[42] Kim E., Choi W., Ryu S., Yun Y., Jo S., Yoo J. (2023). Effect of 3D Lithiophilic Current Collector for Anode-Free Li Ion Batteries. J. Alloys Compd..

[43] Zheng H., Cheng X., Zheng Q., Zhang J., Li T., Xie E., Xu Y. (2023). The Thermodynamically Directed Dendrite-Free Lithium Metal Batteries on LiZn Alloy Surface. Nano Res..

[44] Zeng S.-Y., Wang W.-L., Li D., Yang C., Zheng Z.-J. (2024). Stable Ultrathin Lithium Metal Anode Enabled by Self-Adapting Electrochemical Regulating Strategy. Energy Mater..

[45] Hui Y., Wu Y., Sun W., Sun X., Huang G., Na Z., Hui Y., Wu Y., Sun W., Sun X. (2023). Nanosecond Pulsed Laser-Assisted Deposition to Construct a 3D Quasi-Gradient Lithiophilic Skeleton for Stable Lithium Metal Anodes. Adv. Funct. Mater..

[46] Li C., Yang C., Huang T., Wang Y., Yang J., Jiang Y., Mao J., Zheng S., Xia S., Li C. (2024). In Situ Interphasial Engineering Enabling High-Rate and Long-Cycling Li Metal Batteries. Adv. Funct. Mater..

[47] Bae J., Oh S., Lee B., Lee C.H., Chung J., Kim J., Jo S., Seo S., Lim J., Chung S. (2023). High-Performance, Printable Quasi-Solid-State Electrolytes toward All 3D Direct Ink Writing of Shape-Versatile Li-Ion Batteries. Energy Storage Mater..

[48] Tao R., Gu Y., Sharma J., Hong K., Li J. (2023). A Conformal Heat-Drying Direct Ink Writing 3D Printing for High-Performance Lithium-Ion Batteries. Mater. Today Chem..

[49] Xu D., Zhou N., Wang A., Xu Y., Liu X., Tang S., Luo J. (2023). Mechano-Electrochemically Promoting Lithium Atom Diffusion and Relieving Accumulative Stress for Deep-Cycling Lithium Metal Anodes. Adv. Mater..

[50] Hu X., Gao Y., Sun Y., Hou Z., Luo Y., Wang D., Wang J., Zhang B., Zheng Z., Li Q. (2024). Preserving the Li {110} Texture to Achieve Long Cycle Life in Li Metal Electrode at High Rates. Adv. Funct. Mater..

[51] Tan J., Ma L., Yi P., Wang Y., Li Z., Fang Z., Li X., He S., Wang X., Ye M. (2024). Scalable Customization of Crystallographic Plane Controllable Lithium Metal Anodes for Ultralong-Lasting Lithium Metal Batteries. Adv. Mater..

[52] Kriegler J., Ballmes H., Dib S., Demir A.G., Hille L., Liang Y., Wach L., Weinmann S., Keilhofer J., Kim K.J. (2024). Surface Reconditioning of Lithium Metal Electrodes by Laser Treatment for the Industrial Production of Enhanced Lithium Metal Batteries. Adv. Funct. Mater..

[53] Nogales P.M., Song H.Y., Jo M.H., Jeong S.K. (2022). Improvement in the Electrochemical Properties of Lithium Metal by Heat Treatment: Changes in the Chemical Composition of Native and Solid Electrolyte Interphase Films. Energies.

[54] Liu T., Liu Y., Zhang Y., Zhang L. (2023). Surface Modified by SnI_2_ Boosts Dendrite-Free All-Solid-State Lithium Metal Batteries. J. Electroanal. Chem..

[55] Zou P., Jiang W., Ma L., Ouyang L. (2024). Highly Reversible Lithium Metal Anodes Enabled by a Lithium Sulfamate Layer with High Ionic Conductivity and a Low Surface Diffusion Barrier. J. Mater. Chem. A.

[56] Yang M., Zhang R., Shi C., Liu E., Zhao N. (2023). Ultra-Long Cycle Life of Lithium Metal Anode Achieved by Fluoride Silane Artificial Layer. Mater. Lett..

[57] Tan L., Chen Q., Chen P., Huang X., Li L., Zou K., Liu D. (2023). Lithium Chloride Protective Layer for Stable Lithium Metal Anode via a Facile Surface Chemistry. J. Electroanal. Chem..

[58] Krauskopf B., Otto S.-K., Moryson Y., Hoffmann F., Sann J., Janek J. (2023). Thin and Homogenous Surface Functionalization of Lithium Metal Anodes by Defined Molecular Treatment. J. Electrochem. Soc..

[59] Xie X., Chen J., Chen X., Shi Z. (2023). Exploring the Effect of Lithium Halide Artificial SEI on the Electrochemical Performance of Lithium Metal Batteries. J. Electroanal. Chem..

[60] Yao X., Wang J., Lin S., Tao C., Zhang X., Wang W., Zhao C., Wang L., Bao J.L., Wang Y. (2023). Surface Bromination of Lithium-Metal Anode for High Cyclic Efficiency. Adv. Energy Mater..

[61] Huang D., Zeng C., Liu M., Chen X., Li Y., Hu S., Pan Q., Zheng F., Li Q., Wang H. (2023). Introducing KI as a Functional Electrolyte Additive to Stabilize Li Metal Anode. Chem. Eng. J..

[62] Zhao Y., Zhou T., Jeurgens L.P.H., Kong X., Choi J.W., Coskun A. (2023). Electrolyte Engineering for Highly Inorganic Solid Electrolyte Interphase in High-Performance Lithium Metal Batteries. Chem.

[63] Nogales P.M., Lee S., Yang S., Jeong S.-K. (2024). Effects of Electrolyte Solvent Composition on Solid Electrolyte Interphase Properties in Lithium Metal Batteries: Focusing on Ethylene Carbonate to Ethyl Methyl Carbonate Ratios. Batteries.

[64] Fasulo F., Muñoz-García A.B., Massaro A., Crescenzi O., Huang C., Pavone M. (2023). Vinylene Carbonate Reactivity at Lithium Metal Surface: First-Principles Insights into the Early Steps of SEI Formation. J. Mater. Chem. A.

[65] Tao C., Zheng T., Jia P., Gong W., Yila G., Wang L., Liu T. (2024). Synergy of Weakly Solvated Electrolyte and LiF-Reinforced Interphase Enables Long-Term Operation of Li-Metal Batteries at Low Temperatures. ACS Appl. Mater. Interfaces.

[66] Wen Z., Fang W., Wang F., Kang H., Zhao S., Guo S., Chen G. (2024). Dual-Salt Electrolyte Additive Enables High Moisture Tolerance and Favorable Electric Double Layer for Lithium Metal Battery. Angew. Chem.-Int. Ed..

[67] Zhang Y., Gou Z., Zheng K., Dou Y., Zhou Z. (2024). Enhancing the Stability of Metallic Li Anodes for Aprotic Li-O_2_ Batteries with Dual-Anion Electrolytes. J. Phys. Chem. Lett..

[68] Park M., Ha S., Park J., Kang D.H., Hyun J.C., Yoon J., Jin H.J., Yun Y.S. (2023). Multifunctional Surface-Engineering of 3D-Lithiophilic Nanocarbon Scaffold for High-Voltage Anode-Minimized Lithium Metal Batteries. Chem. Eng. J..

[69] Sun Z., Zhang Q., Wang Z., Chen Y., Wang K., Shen F., Guo J., Han X. (2024). Guiding Lithium Growth Direction by Au Coated Separator for Improving Lithium Metal Anode. Energy Mater..

[70] Xiong X., Yan W., Zhu Y., Liu L., Fu L., Chen Y., Yu N., Wu Y., Wang B., Xiao R. (2022). Li_4_Ti_5_O_12_ Coating on Copper Foil as Ion Redistributor Layer for Stable Lithium Metal Anode. Adv. Energy Mater..

[71] Peng B., Liu Z., Zhou Q., Xiong X., Xia S., Yuan X., Wang F., Ozoemena K.I., Liu L., Fu L. (2024). A Solid-State Electrolyte Based on Li_0.95_Na_0.05_FePO_4_ for Lithium Metal Batteries. Adv. Mater..

